# *rac*-(*Z*)-Methyl 1-benzyl-3-[(3-hy­droxy­quinuclidin-2-yl­idene)meth­yl]-1*H*-indole-6-carboxyl­ate

**DOI:** 10.1107/S1600536812040731

**Published:** 2012-10-13

**Authors:** Narsimha Reddy Penthala, Purushotham Rao Ponugoti, Sean Parkin, Peter A. Crooks

**Affiliations:** a Dept. of Pharm. Sciences, College of Pharmacy, University of Arkansas for Medical Sciences, Little Rock, AR 72205, USA; b Dept. of Chemistry, University of Kentucky, Lexington KY 40506, USA

## Abstract

In the title compound, C_25_H_26_N_2_O_3_, the double bond connecting the aza-bicyclic and indole units has *Z* geometry. The compound was obtained as a racemate, and since the crystal is centrosymmetric it contains equal amounts of the *S* and *R* enanti­omers. However, the structure is disordered such that the asymmetric unit contains both enanti­omers in unequal amounts [refined occupancies 0.904 (2) and 0.096 (2)]. The dihedral angle between the benzene ring of the benzyl group and the mean plane of the indole ring is 76.07 (3) °. In the crystal, mol­ecules are linked by O—H⋯O_carbon­yl_ hydrogen bonds into chains propagating in [110].

## Related literature

For background literature, see: Sekhar *et al.*, (2003 ▶), Amudhan *et al.*, (2010 ▶). For the biological activity of *N*-benzyl indole quinuclidinone, see: Sonar *et al.* (2007 ▶). For a similar crystal structure, see: Sonar *et al.*, (2004 ▶).
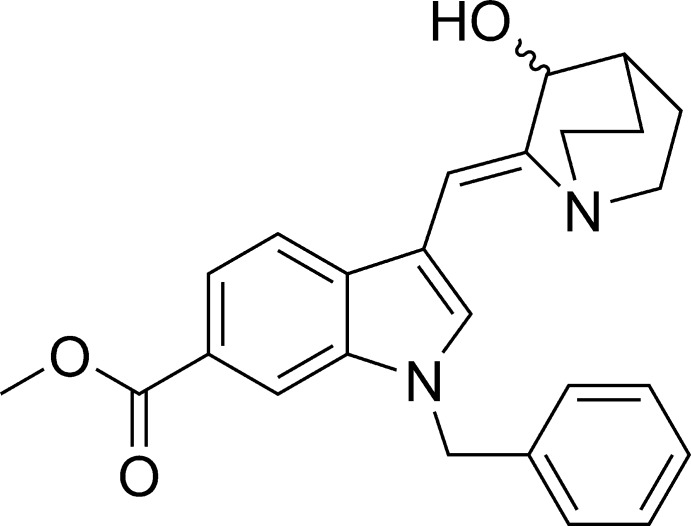


## Experimental

### 

#### Crystal data

C_25_H_26_N_2_O_3_
*M* *_r_* = 402.48
Triclinic, 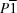

*a* = 6.1619 (1) Å
*b* = 10.3119 (2) Å
*c* = 16.5959 (3) Å
α = 75.6705 (7)°
β = 80.0939 (7)°
γ = 85.9410 (7)°
*V* = 1006.04 (3) Å^3^
*Z* = 2
Mo *K*α radiation
μ = 0.09 mm^−1^
*T* = 90 K
0.28 × 0.22 × 0.12 mm

#### Data collection

Nonius KappaCCD diffractometer
9126 measured reflections
4597 independent reflections
3831 reflections with *I* > 2σ(*I*)
*R*_int_ = 0.024

#### Refinement

*R*[*F* ^2^ > 2σ(*F* ^2^)] = 0.039
*wR*(*F* ^2^) = 0.102
*S* = 1.04
4597 reflections
312 parameters
64 restraints
H-atom parameters constrained
Δρ_max_ = 0.27 e Å^−3^
Δρ_min_ = −0.20 e Å^−3^

### 

Data collection: *COLLECT* (Nonius, 1998 ▶); cell refinement: *SCALEPACK* (Otwinowski & Minor, 1997 ▶); data reduction: *DENZO-SMN* (Otwinowski & Minor, 1997 ▶); program(s) used to solve structure: *SHELXS97* (Sheldrick, 2008 ▶); program(s) used to refine structure: *SHELXL97* (Sheldrick, 2008 ▶); molecular graphics: *XP* in *SHELXTL* (Sheldrick, 2008 ▶); software used to prepare material for publication: *SHELXL97* and local procedures.

## Supplementary Material

Crystal structure: contains datablock(s) global, I. DOI: 10.1107/S1600536812040731/hg5242sup1.cif

Structure factors: contains datablock(s) I. DOI: 10.1107/S1600536812040731/hg5242Isup2.hkl

Additional supplementary materials:  crystallographic information; 3D view; checkCIF report

## Figures and Tables

**Table 1:** Hydrogen-bond geometry (Å, °)

*D*—H⋯*A*	*D*—H	H⋯*A*	*D*⋯*A*	*D*—H⋯*A*
O1—H1*A*⋯O2^i^	0.84	1.97	2.7989 (13)	169

Symmetry code: (i) 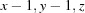.

**Fig. 1.**
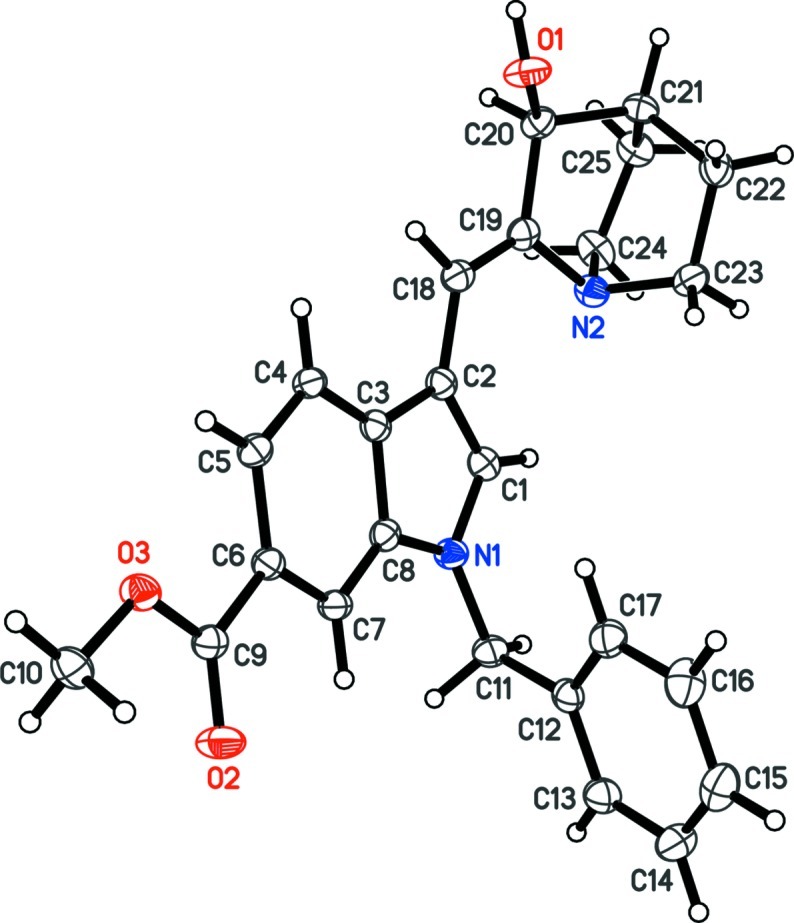
A view of the molecule with displacement ellipsoids drawn at the 50% probability level.

## supplementary crystallographic information

### Comment

The *N*-benzyl indole quinuclidin-3-one moiety has been found to possess interesting biological properties which range from NADPH oxidase activity (Sekhar *et al.*, 2003), thermal sensitizing activity to radiation treatment (Sonar *et al.*, 2007), and also exhibit antiangiogenic properties (Amudhan *et al.*, 2010). Previously, we reported the crystal structure of *Z*-(*S*)-2-(1- phenylsulfonyl-1*H*-indol-3-ylmethylene)-1-azabicyclo[2.2.2]octan-3-ol (Sonar *et al.*, 2004) and in continuation of our research work on the *N*-benzyl indole quinuclidin-3-one scaffold (Sonar *et al.*, 2007) we designed and synthesized a novel series of carboxymethyl *N*-benzyl indole quinuclidin-3-ones as potent anticancer agents. The title compound was prepared by the reduction of *Z*-methyl 1-benzyl-3-[(3-oxoquinuclidin-2-ylidene)methyl]-1*H*-indole-6-carboxylate with NaBH_4_ in methanol at room temperature (Sonar *et al.*, 2004). Recrystallization of the title compound from methanol afforded colorless needles that were suitable for X-ray analysis.

The X-ray studies revealed that the title compound is the *Z* isomer having the C2—C18 bond in a *trans* position with respect to the C19—C20 bond. The double bond (C18═C19) has a nearly planar atomic arrangement, since the r.m.s. deviation from the best plane passing through atoms N2, C19, C20, C18 and C2 is 0.265 (1) Å. In this molecule, the azabicyclic system presents very small distortions around atoms N2, C24, C21, C22, C23 and C20. The value of the C1—C2—C18—C19 torsion angle [-5.1 (2)°] indicates the deviation of the indole ring from the plane of the double bond connected to the azabicyclic ring. The dihedral angle between the benzene ring of the benzyl group and with the mean plane of the indole ring is 76.07 (3) Å. The crystal of the title compound is a racemic equimolar mixture of *R* and *S* configurations. However, the structure is disordered such that the asymmetric unit contains both enantiomers, but in unequal amounts [refined occupancies 0.904 (2) and 0.096 (2)]. The hydrogen atom of the disordered OH group is involved in an intermolecular hydrogen bond with the carbonyl oxygen O2 on an adjacent molecule, thus forming an infinite chain-like structure along the (110) direction.

### Experimental

The compound *Z*-methyl 1-benzyl-3-[(3-oxoquinuclidin-2-ylidene)methyl] -1*H*-indole-6-carboxylate was prepared by the available literature procedure (Sonar *et al.*, 2004). To a stirred solution of *Z*-methyl 1-benzyl-3-((3-oxoquinuclidin-2-ylidene)methyl)-1*H*-indole-6-carboxylate (0.40 g, 1 mmol) in methanol (15 ml) at 273 K was added NaBH_4_ (0.38 g, 10 mmol) over a period of 15 min and stirring was continued for 2 h at room temperature. Water (50 ml) was added and the mixture was extracted with CHCl_3_ (3 times 10 ml). The combined organic layers were dried over Na_2_SO_4_ and evaporated to give the title compound as a white solid. Recrystallization from methanol afforded colorless needles suitable for X-ray analysis.

### Refinement

H atoms were found in difference Fourier maps and subsequently placed in idealized positions with constrained distances of 0.98 Å (RCH_3_), 0.99 Å (*R*_2_CH_2_), 1.00 Å (*R*_3_CH), 0.95 Å (C_sp2_H), 0.84 Å (O—H), and with *U*_iso_(H) values set to either 1.2*U*_eq_ or 1.5*U*_eq_ (RCH_3_, OH) of the attached atom.

To ensure satisfactory refinement of the disordered parts of the structure, restraints were needed. The restraints (*SHELXL97* commands SAME, DELU, SAME) were used to ensure similar geometries and displacement parameters of closely proximate, chemically similar groups.

### Crystal data

**Table:**

C_25_H_26_N_2_O_3_	*Z* = 2
*M**_r_* = 402.48	*F*(000) = 428
Triclinic, *P*1	*D*_x_ = 1.329 Mg m^−^^3^
Hall symbol: -P 1	Mo *K*α radiation, λ = 0.71073 Å
*a* = 6.1619 (1) Å	Cell parameters from 4571 reflections
*b* = 10.3119 (2) Å	θ = 1.0–27.5°
*c* = 16.5959 (3) Å	µ = 0.09 mm^−^^1^
α = 75.6705 (7)°	*T* = 90 K
β = 80.0939 (7)°	Block, yellow
γ = 85.9410 (7)°	0.28 × 0.22 × 0.12 mm
*V* = 1006.04 (3) Å^3^	

### Data collection

**Table:**

Nonius KappaCCD diffractometer	3831 reflections with *I* > 2σ(*I*)
Radiation source: fine-focus sealed tube	*R*_int_ = 0.024
Graphite monochromator	θ_max_ = 27.5°, θ_min_ = 2.0°
Detector resolution: 9.1 pixels mm^-1^	*h* = −8→8
ω scans at fixed χ = 55°	*k* = −13→13
9126 measured reflections	*l* = −21→21
4597 independent reflections	

### Refinement

**Table:**

Refinement on *F*^2^	Secondary atom site location: difference Fourier map
Least-squares matrix: full	Hydrogen site location: inferred from neighbouring sites
*R*[*F*^2^ > 2σ(*F*^2^)] = 0.039	H-atom parameters constrained
*wR*(*F*^2^) = 0.102	*w* = 1/[σ^2^(*F*_o_^2^) + (0.0442*P*)^2^ + 0.3522*P*] where *P* = (*F*_o_^2^ + 2*F*_c_^2^)/3
*S* = 1.04	(Δ/σ)_max_ = 0.001
4597 reflections	Δρ_max_ = 0.27 e Å^−^^3^
312 parameters	Δρ_min_ = −0.20 e Å^−^^3^
64 restraints	Extinction correction: *SHELXL*, Fc^*^=kFc[1+0.001xFc^2^λ^3^/sin(2θ)]^-1/4^
Primary atom site location: structure-invariant direct methods	Extinction coefficient: 0.023 (4)

### Special details

**Table:**

Geometry. All e.s.d.'s (except the e.s.d. in the dihedral angle between two l.s. planes) are estimated using the full covariance matrix. The cell e.s.d.'s are taken into account individually in the estimation of e.s.d.'s in distances, angles and torsion angles; correlations between e.s.d.'s in cell parameters are only used when they are defined by crystal symmetry. An approximate (isotropic) treatment of cell e.s.d.'s is used for estimating e.s.d.'s involving l.s. planes.
Refinement. Refinement of *F*^2^ against all reflections. The weighted *R*-value *wR* and goodness of fit *S* are based on *F*^2^. Conventional *R*-values *R* are based on *F*, with *F* set to zero for negative *F*^2^. The threshold expression of *F*^2^ > 2σ(*F*^2^) is used only for calculating *R*-factors(gt) *etc*. and is not relevant to the choice of reflections for refinement. *R*-values based on *F*^2^ are statistically about twice as large as those based on *F*, and *R*-values based on ALL data will be even larger.

### Fractional atomic coordinates and isotropic or equivalent isotropic displacement parameters (Å^2^)

**Table:**

	*x*	*y*	*z*	*U*_iso_*/*U*_eq_	Occ. (<1)
N1	0.31767 (16)	0.66937 (10)	0.24130 (6)	0.0190 (2)	
O2	1.05912 (15)	0.90627 (9)	0.09629 (6)	0.0280 (2)	
O3	1.17472 (13)	0.78520 (8)	0.00096 (5)	0.0213 (2)	
C1	0.1919 (2)	0.56139 (12)	0.24673 (7)	0.0196 (2)	
H1	0.0613	0.5379	0.2861	0.024*	
C2	0.28163 (19)	0.49219 (11)	0.18719 (7)	0.0181 (2)	
C3	0.47803 (19)	0.56153 (11)	0.14234 (7)	0.0172 (2)	
C4	0.64453 (19)	0.53937 (11)	0.07749 (7)	0.0180 (2)	
H4	0.6356	0.4677	0.0517	0.022*	
C5	0.82107 (19)	0.62300 (11)	0.05167 (7)	0.0182 (2)	
H5	0.9329	0.6092	0.0072	0.022*	
C6	0.83869 (19)	0.72871 (11)	0.08999 (7)	0.0171 (2)	
C7	0.67658 (19)	0.75346 (11)	0.15415 (7)	0.0181 (2)	
H7	0.6879	0.8245	0.1802	0.022*	
C8	0.49701 (19)	0.67018 (11)	0.17875 (7)	0.0171 (2)	
C9	1.03202 (19)	0.81587 (11)	0.06408 (7)	0.0184 (2)	
C10	1.3663 (2)	0.86728 (12)	−0.02905 (8)	0.0230 (3)	
H10A	1.3191	0.9616	−0.0455	0.035*	
H10B	1.4539	0.8408	−0.0779	0.035*	
H10C	1.4557	0.8552	0.0159	0.035*	
C11	0.2901 (2)	0.75327 (12)	0.30102 (8)	0.0212 (3)	
H11A	0.1373	0.7463	0.3319	0.025*	
H11B	0.3117	0.8477	0.2696	0.025*	
C12	0.4496 (2)	0.71600 (12)	0.36448 (7)	0.0196 (3)	
C13	0.4619 (2)	0.79843 (13)	0.41841 (8)	0.0248 (3)	
H13	0.3700	0.8769	0.4156	0.030*	
C14	0.6069 (2)	0.76729 (14)	0.47622 (8)	0.0302 (3)	
H14	0.6149	0.8250	0.5122	0.036*	
C15	0.7402 (2)	0.65246 (15)	0.48175 (8)	0.0307 (3)	
H15	0.8390	0.6309	0.5215	0.037*	
C16	0.7282 (2)	0.56959 (14)	0.42883 (8)	0.0300 (3)	
H16	0.8186	0.4905	0.4325	0.036*	
C17	0.5845 (2)	0.60137 (13)	0.37025 (8)	0.0250 (3)	
H17	0.5786	0.5441	0.3338	0.030*	
C18	0.2112 (2)	0.36924 (12)	0.17230 (7)	0.0203 (3)	
H18	0.2905	0.3426	0.1244	0.024*	
C19	0.0490 (3)	0.28894 (14)	0.21750 (9)	0.0190 (3)	0.904 (2)
C20	0.0007 (2)	0.15731 (14)	0.19936 (9)	0.0204 (3)	0.904 (2)
H20	−0.0961	0.1753	0.1550	0.025*	0.904 (2)
O1	0.20291 (17)	0.09447 (10)	0.16992 (7)	0.0268 (3)	0.904 (2)
H1A	0.1766	0.0378	0.1445	0.040*	0.904 (2)
C21	−0.1226 (3)	0.07182 (18)	0.28158 (11)	0.0203 (4)	0.904 (2)
H21	−0.1372	−0.0216	0.2763	0.024*	0.904 (2)
C22	0.0068 (3)	0.07145 (14)	0.35275 (9)	0.0237 (3)	0.904 (2)
H22A	0.1614	0.0414	0.3378	0.028*	0.904 (2)
H22B	−0.0598	0.0092	0.4054	0.028*	0.904 (2)
C23	−0.0005 (3)	0.2159 (2)	0.36551 (10)	0.0232 (3)	0.904 (2)
H23A	−0.0995	0.2201	0.4186	0.028*	0.904 (2)
H23B	0.1488	0.2397	0.3703	0.028*	0.904 (2)
N2	−0.0804 (3)	0.31334 (13)	0.29406 (9)	0.0209 (3)	0.904 (2)
C24	−0.3148 (3)	0.28641 (16)	0.29584 (10)	0.0258 (4)	0.904 (2)
H24A	−0.3657	0.3432	0.2447	0.031*	0.904 (2)
H24B	−0.4054	0.3110	0.3455	0.031*	0.904 (2)
C25	−0.3496 (2)	0.13790 (14)	0.29981 (9)	0.0243 (3)	0.904 (2)
H25A	−0.4259	0.0929	0.3563	0.029*	0.904 (2)
H25B	−0.4408	0.1311	0.2575	0.029*	0.904 (2)
C19'	0.116 (2)	0.2718 (12)	0.2322 (7)	0.013 (3)*	0.096 (2)
C20'	0.080 (3)	0.1336 (14)	0.2189 (9)	0.033 (4)*	0.096 (2)
H20'	0.1935	0.0690	0.2434	0.039*	0.096 (2)
O1'	0.1065 (19)	0.1428 (11)	0.1316 (6)	0.035 (3)*	0.096 (2)
H1'	0.0220	0.0896	0.1222	0.052*	0.096 (2)
C21'	−0.148 (3)	0.0902 (19)	0.2671 (11)	0.039 (5)*	0.096 (2)
H21'	−0.1966	0.0125	0.2494	0.047*	0.096 (2)
C22'	−0.124 (3)	0.0512 (14)	0.3604 (9)	0.036 (4)*	0.096 (2)
H22C	−0.2711	0.0409	0.3964	0.044*	0.096 (2)
H22D	−0.0383	−0.0339	0.3739	0.044*	0.096 (2)
C23'	−0.005 (3)	0.1654 (15)	0.3737 (10)	0.022 (4)*	0.096 (2)
H23C	−0.0666	0.1803	0.4300	0.027*	0.096 (2)
H23D	0.1519	0.1378	0.3743	0.027*	0.096 (2)
N2'	−0.016 (2)	0.2938 (14)	0.3109 (8)	0.021 (3)*	0.096 (2)
C24'	−0.240 (3)	0.3146 (15)	0.2931 (10)	0.022 (4)*	0.096 (2)
H24C	−0.2543	0.4049	0.2558	0.026*	0.096 (2)
H24D	−0.3418	0.3118	0.3465	0.026*	0.096 (2)
C25'	−0.310 (2)	0.2096 (15)	0.2506 (10)	0.036 (4)*	0.096 (2)
H25C	−0.4629	0.1816	0.2752	0.043*	0.096 (2)
H25D	−0.3038	0.2475	0.1893	0.043*	0.096 (2)

### Atomic displacement parameters (Å^2^)

**Table:**

	*U*^11^	*U*^22^	*U*^33^	*U*^12^	*U*^13^	*U*^23^
N1	0.0196 (5)	0.0182 (5)	0.0198 (5)	−0.0002 (4)	−0.0017 (4)	−0.0068 (4)
O2	0.0274 (5)	0.0277 (5)	0.0337 (5)	−0.0079 (4)	−0.0019 (4)	−0.0166 (4)
O3	0.0178 (4)	0.0223 (4)	0.0242 (4)	−0.0048 (3)	−0.0004 (3)	−0.0073 (3)
C1	0.0183 (6)	0.0188 (6)	0.0211 (6)	−0.0006 (4)	−0.0034 (5)	−0.0034 (5)
C2	0.0179 (6)	0.0178 (5)	0.0182 (5)	−0.0005 (4)	−0.0045 (4)	−0.0027 (4)
C3	0.0180 (6)	0.0162 (5)	0.0174 (5)	0.0000 (4)	−0.0057 (4)	−0.0023 (4)
C4	0.0208 (6)	0.0173 (5)	0.0177 (5)	−0.0005 (4)	−0.0047 (4)	−0.0061 (4)
C5	0.0176 (6)	0.0194 (6)	0.0182 (5)	0.0007 (4)	−0.0028 (4)	−0.0058 (4)
C6	0.0177 (6)	0.0165 (5)	0.0178 (5)	−0.0008 (4)	−0.0060 (4)	−0.0033 (4)
C7	0.0209 (6)	0.0156 (5)	0.0192 (6)	0.0006 (4)	−0.0063 (5)	−0.0055 (4)
C8	0.0181 (6)	0.0169 (5)	0.0163 (5)	0.0018 (4)	−0.0039 (4)	−0.0038 (4)
C9	0.0186 (6)	0.0181 (6)	0.0194 (6)	0.0008 (4)	−0.0061 (5)	−0.0048 (4)
C10	0.0173 (6)	0.0235 (6)	0.0273 (6)	−0.0051 (5)	−0.0028 (5)	−0.0037 (5)
C11	0.0233 (6)	0.0190 (6)	0.0223 (6)	0.0025 (5)	−0.0014 (5)	−0.0088 (5)
C12	0.0206 (6)	0.0184 (6)	0.0179 (5)	−0.0025 (4)	0.0014 (4)	−0.0034 (4)
C13	0.0289 (7)	0.0210 (6)	0.0245 (6)	−0.0008 (5)	−0.0021 (5)	−0.0070 (5)
C14	0.0375 (8)	0.0316 (7)	0.0244 (7)	−0.0059 (6)	−0.0052 (6)	−0.0106 (5)
C15	0.0287 (7)	0.0395 (8)	0.0236 (6)	−0.0015 (6)	−0.0069 (5)	−0.0052 (6)
C16	0.0288 (7)	0.0320 (7)	0.0263 (7)	0.0078 (6)	−0.0034 (5)	−0.0049 (6)
C17	0.0300 (7)	0.0230 (6)	0.0218 (6)	0.0029 (5)	−0.0030 (5)	−0.0069 (5)
C18	0.0220 (6)	0.0209 (6)	0.0191 (6)	−0.0019 (5)	−0.0033 (5)	−0.0062 (5)
C19	0.0175 (7)	0.0211 (7)	0.0195 (7)	0.0005 (6)	−0.0064 (6)	−0.0048 (5)
C20	0.0179 (7)	0.0246 (7)	0.0207 (7)	−0.0037 (5)	−0.0028 (6)	−0.0082 (5)
O1	0.0232 (5)	0.0290 (6)	0.0325 (6)	−0.0072 (4)	0.0033 (4)	−0.0194 (5)
C21	0.0188 (7)	0.0180 (7)	0.0250 (8)	−0.0030 (5)	−0.0025 (6)	−0.0066 (6)
C22	0.0238 (8)	0.0230 (7)	0.0233 (7)	0.0025 (6)	−0.0058 (6)	−0.0035 (5)
C23	0.0242 (8)	0.0260 (9)	0.0215 (8)	−0.0027 (7)	−0.0035 (5)	−0.0095 (7)
N2	0.0193 (8)	0.0196 (6)	0.0230 (7)	−0.0018 (5)	−0.0001 (6)	−0.0055 (5)
C24	0.0156 (8)	0.0233 (8)	0.0344 (9)	0.0021 (6)	−0.0008 (6)	−0.0025 (6)
C25	0.0174 (7)	0.0251 (7)	0.0302 (8)	−0.0037 (5)	−0.0030 (5)	−0.0059 (6)

### Geometric parameters (Å, º)

**Table:**

N1—C1	1.3772 (15)	C19—C20	1.5204 (18)
N1—C8	1.3787 (15)	C20—O1	1.4296 (18)
N1—C11	1.4510 (15)	C20—C21	1.536 (2)
O2—C9	1.2174 (14)	C20—H20	1.0000
O3—C9	1.3334 (14)	O1—H1A	0.8400
O3—C10	1.4450 (14)	C21—C25	1.528 (2)
C1—C2	1.3771 (17)	C21—C22	1.533 (2)
C1—H1	0.9500	C21—H21	1.0000
C2—C3	1.4419 (16)	C22—C23	1.552 (2)
C2—C18	1.4583 (16)	C22—H22A	0.9900
C3—C4	1.4042 (16)	C22—H22B	0.9900
C3—C8	1.4185 (15)	C23—N2	1.483 (2)
C4—C5	1.3784 (16)	C23—H23A	0.9900
C4—H4	0.9500	C23—H23B	0.9900
C5—C6	1.4100 (15)	N2—C24	1.484 (2)
C5—H5	0.9500	C24—C25	1.545 (2)
C6—C7	1.3890 (16)	C24—H24A	0.9900
C6—C9	1.4819 (16)	C24—H24B	0.9900
C7—C8	1.3907 (16)	C25—H25A	0.9900
C7—H7	0.9500	C25—H25B	0.9900
C10—H10A	0.9800	C19'—N2'	1.473 (13)
C10—H10B	0.9800	C19'—C20'	1.532 (14)
C10—H10C	0.9800	C20'—O1'	1.410 (14)
C11—C12	1.5245 (17)	C20'—C21'	1.525 (16)
C11—H11A	0.9900	C20'—H20'	1.0000
C11—H11B	0.9900	O1'—H1'	0.8400
C12—C17	1.3894 (17)	C21'—C22'	1.530 (16)
C12—C13	1.3924 (17)	C21'—C25'	1.534 (16)
C13—C14	1.3871 (19)	C21'—H21'	1.0000
C13—H13	0.9500	C22'—C23'	1.511 (15)
C14—C15	1.386 (2)	C22'—H22C	0.9900
C14—H14	0.9500	C22'—H22D	0.9900
C15—C16	1.382 (2)	C23'—N2'	1.474 (14)
C15—H15	0.9500	C23'—H23C	0.9900
C16—C17	1.3906 (18)	C23'—H23D	0.9900
C16—H16	0.9500	N2'—C24'	1.449 (14)
C17—H17	0.9500	C24'—C25'	1.552 (15)
C18—C19'	1.317 (11)	C24'—H24C	0.9900
C18—C19	1.3414 (18)	C24'—H24D	0.9900
C18—H18	0.9500	C25'—H25C	0.9900
C19—N2	1.4473 (17)	C25'—H25D	0.9900
			
C1—N1—C8	108.58 (10)	C25—C21—C20	107.91 (15)
C1—N1—C11	125.67 (10)	C22—C21—C20	108.15 (13)
C8—N1—C11	124.84 (10)	C25—C21—H21	110.5
C9—O3—C10	116.16 (9)	C22—C21—H21	110.5
C2—C1—N1	110.67 (11)	C20—C21—H21	110.5
C2—C1—H1	124.7	C21—C22—C23	108.31 (12)
N1—C1—H1	124.7	C21—C22—H22A	110.0
C1—C2—C3	105.81 (10)	C23—C22—H22A	110.0
C1—C2—C18	129.80 (11)	C21—C22—H22B	110.0
C3—C2—C18	124.28 (11)	C23—C22—H22B	110.0
C4—C3—C8	118.63 (10)	H22A—C22—H22B	108.4
C4—C3—C2	134.05 (11)	N2—C23—C22	110.86 (12)
C8—C3—C2	107.24 (10)	N2—C23—H23A	109.5
C5—C4—C3	119.14 (10)	C22—C23—H23A	109.5
C5—C4—H4	120.4	N2—C23—H23B	109.5
C3—C4—H4	120.4	C22—C23—H23B	109.5
C4—C5—C6	121.22 (11)	H23A—C23—H23B	108.1
C4—C5—H5	119.4	C19—N2—C23	107.06 (13)
C6—C5—H5	119.4	C19—N2—C24	109.12 (13)
C7—C6—C5	121.04 (10)	C23—N2—C24	108.13 (12)
C7—C6—C9	117.76 (10)	N2—C24—C25	112.14 (12)
C5—C6—C9	121.20 (10)	N2—C24—H24A	109.2
C6—C7—C8	117.38 (10)	C25—C24—H24A	109.2
C6—C7—H7	121.3	N2—C24—H24B	109.2
C8—C7—H7	121.3	C25—C24—H24B	109.2
N1—C8—C7	129.70 (10)	H24A—C24—H24B	107.9
N1—C8—C3	107.67 (10)	C21—C25—C24	107.59 (12)
C7—C8—C3	122.56 (11)	C21—C25—H25A	110.2
O2—C9—O3	123.10 (11)	C24—C25—H25A	110.2
O2—C9—C6	124.16 (11)	C21—C25—H25B	110.2
O3—C9—C6	112.74 (9)	C24—C25—H25B	110.2
O3—C10—H10A	109.5	H25A—C25—H25B	108.5
O3—C10—H10B	109.5	C18—C19'—N2'	123.0 (10)
H10A—C10—H10B	109.5	C18—C19'—C20'	123.3 (9)
O3—C10—H10C	109.5	N2'—C19'—C20'	112.0 (9)
H10A—C10—H10C	109.5	O1'—C20'—C21'	114.7 (13)
H10B—C10—H10C	109.5	O1'—C20'—C19'	108.5 (11)
N1—C11—C12	113.36 (10)	C21'—C20'—C19'	107.0 (12)
N1—C11—H11A	108.9	O1'—C20'—H20'	108.8
C12—C11—H11A	108.9	C21'—C20'—H20'	108.8
N1—C11—H11B	108.9	C19'—C20'—H20'	108.8
C12—C11—H11B	108.9	C20'—O1'—H1'	109.5
H11A—C11—H11B	107.7	C20'—C21'—C22'	106.2 (13)
C17—C12—C13	118.55 (11)	C20'—C21'—C25'	108.6 (14)
C17—C12—C11	121.83 (11)	C22'—C21'—C25'	109.8 (14)
C13—C12—C11	119.62 (11)	C20'—C21'—H21'	110.7
C14—C13—C12	120.72 (12)	C22'—C21'—H21'	110.7
C14—C13—H13	119.6	C25'—C21'—H21'	110.7
C12—C13—H13	119.6	C23'—C22'—C21'	104.9 (12)
C15—C14—C13	120.31 (12)	C23'—C22'—H22C	110.8
C15—C14—H14	119.8	C21'—C22'—H22C	110.8
C13—C14—H14	119.8	C23'—C22'—H22D	110.8
C16—C15—C14	119.39 (12)	C21'—C22'—H22D	110.8
C16—C15—H15	120.3	H22C—C22'—H22D	108.8
C14—C15—H15	120.3	N2'—C23'—C22'	116.0 (12)
C15—C16—C17	120.38 (13)	N2'—C23'—H23C	108.3
C15—C16—H16	119.8	C22'—C23'—H23C	108.3
C17—C16—H16	119.8	N2'—C23'—H23D	108.3
C12—C17—C16	120.65 (12)	C22'—C23'—H23D	108.3
C12—C17—H17	119.7	H23C—C23'—H23D	107.4
C16—C17—H17	119.7	C24'—N2'—C23'	107.8 (12)
C19'—C18—C2	123.8 (5)	C24'—N2'—C19'	104.4 (11)
C19—C18—C2	128.40 (12)	C23'—N2'—C19'	105.6 (11)
C19'—C18—H18	115.1	N2'—C24'—C25'	113.8 (11)
C19—C18—H18	115.8	N2'—C24'—H24C	108.8
C2—C18—H18	115.8	C25'—C24'—H24C	108.8
C18—C19—N2	122.23 (12)	N2'—C24'—H24D	108.8
C18—C19—C20	123.71 (12)	C25'—C24'—H24D	108.8
N2—C19—C20	113.72 (11)	H24C—C24'—H24D	107.7
O1—C20—C19	109.43 (11)	C21'—C25'—C24'	106.2 (12)
O1—C20—C21	112.52 (13)	C21'—C25'—H25C	110.5
C19—C20—C21	106.93 (11)	C24'—C25'—H25C	110.5
O1—C20—H20	109.3	C21'—C25'—H25D	110.5
C19—C20—H20	109.3	C24'—C25'—H25D	110.5
C21—C20—H20	109.3	H25C—C25'—H25D	108.7
C25—C21—C22	109.07 (13)		
			
C8—N1—C1—C2	−1.61 (13)	C2—C18—C19—C20	−175.48 (13)
C11—N1—C1—C2	−171.05 (10)	C18—C19—C20—O1	35.0 (2)
N1—C1—C2—C3	0.62 (13)	N2—C19—C20—O1	−138.42 (14)
N1—C1—C2—C18	176.88 (11)	C18—C19—C20—C21	157.16 (17)
C1—C2—C3—C4	177.33 (12)	N2—C19—C20—C21	−16.29 (19)
C18—C2—C3—C4	0.8 (2)	O1—C20—C21—C25	−171.58 (11)
C1—C2—C3—C8	0.56 (12)	C19—C20—C21—C25	68.25 (16)
C18—C2—C3—C8	−175.96 (10)	O1—C20—C21—C22	70.56 (15)
C8—C3—C4—C5	0.38 (16)	C19—C20—C21—C22	−49.60 (17)
C2—C3—C4—C5	−176.11 (12)	C25—C21—C22—C23	−51.32 (16)
C3—C4—C5—C6	0.94 (17)	C20—C21—C22—C23	65.79 (16)
C4—C5—C6—C7	−1.10 (17)	C21—C22—C23—N2	−13.47 (17)
C4—C5—C6—C9	178.01 (11)	C18—C19—N2—C23	−104.06 (18)
C5—C6—C7—C8	−0.11 (16)	C20—C19—N2—C23	69.50 (18)
C9—C6—C7—C8	−179.26 (10)	C18—C19—N2—C24	139.14 (17)
C1—N1—C8—C7	−175.07 (12)	C20—C19—N2—C24	−47.30 (18)
C11—N1—C8—C7	−5.52 (19)	C22—C23—N2—C19	−50.65 (16)
C1—N1—C8—C3	1.92 (12)	C22—C23—N2—C24	66.81 (15)
C11—N1—C8—C3	171.47 (10)	C19—N2—C24—C25	64.01 (16)
C6—C7—C8—N1	178.07 (11)	C23—N2—C24—C25	−52.12 (16)
C6—C7—C8—C3	1.47 (16)	C22—C21—C25—C24	65.38 (16)
C4—C3—C8—N1	−178.88 (10)	C20—C21—C25—C24	−51.89 (16)
C2—C3—C8—N1	−1.52 (12)	N2—C24—C25—C21	−12.01 (18)
C4—C3—C8—C7	−1.63 (17)	C19—C18—C19'—N2'	−82.4 (17)
C2—C3—C8—C7	175.73 (10)	C2—C18—C19'—N2'	27.3 (16)
C10—O3—C9—O2	−0.86 (16)	C19—C18—C19'—C20'	81.5 (17)
C10—O3—C9—C6	178.94 (9)	C2—C18—C19'—C20'	−168.7 (10)
C7—C6—C9—O2	1.15 (17)	C18—C19'—C20'—O1'	−16.1 (19)
C5—C6—C9—O2	−177.99 (11)	N2'—C19'—C20'—O1'	149.4 (13)
C7—C6—C9—O3	−178.65 (10)	C18—C19'—C20'—C21'	−140.4 (14)
C5—C6—C9—O3	2.21 (15)	N2'—C19'—C20'—C21'	25.1 (17)
C1—N1—C11—C12	98.55 (13)	O1'—C20'—C21'—C22'	165.5 (13)
C8—N1—C11—C12	−69.23 (14)	C19'—C20'—C21'—C22'	−74.1 (15)
N1—C11—C12—C17	−8.29 (16)	O1'—C20'—C21'—C25'	−76.4 (17)
N1—C11—C12—C13	171.63 (11)	C19'—C20'—C21'—C25'	44.0 (16)
C17—C12—C13—C14	0.46 (19)	C20'—C21'—C22'—C23'	48.1 (17)
C11—C12—C13—C14	−179.46 (12)	C25'—C21'—C22'—C23'	−69.2 (17)
C12—C13—C14—C15	−0.7 (2)	C21'—C22'—C23'—N2'	21 (2)
C13—C14—C15—C16	0.3 (2)	C22'—C23'—N2'—C24'	42.2 (18)
C14—C15—C16—C17	0.3 (2)	C22'—C23'—N2'—C19'	−68.9 (17)
C13—C12—C17—C16	0.21 (19)	C18—C19'—N2'—C24'	92.0 (15)
C11—C12—C17—C16	−179.87 (12)	C20'—C19'—N2'—C24'	−73.5 (15)
C15—C16—C17—C12	−0.6 (2)	C18—C19'—N2'—C23'	−154.4 (13)
C1—C2—C18—C19'	−32.0 (8)	C20'—C19'—N2'—C23'	40.0 (15)
C3—C2—C18—C19'	143.6 (8)	C23'—N2'—C24'—C25'	−64.7 (16)
C1—C2—C18—C19	−5.1 (2)	C19'—N2'—C24'—C25'	47.2 (16)
C3—C2—C18—C19	170.57 (14)	C20'—C21'—C25'—C24'	−67.2 (16)
C19'—C18—C19—N2	83.7 (14)	C22'—C21'—C25'—C24'	48.5 (17)
C2—C18—C19—N2	−2.6 (2)	N2'—C24'—C25'—C21'	18.4 (18)
C19'—C18—C19—C20	−89.2 (14)		

### Hydrogen-bond geometry (Å, º)

**Table:**

*D*—H···*A*	*D*—H	H···*A*	*D*···*A*	*D*—H···*A*
O1—H1*A*···O2^i^	0.84	1.97	2.7989 (13)	169

Symmetry code: (i) *x*−1, *y*−1, *z*.
